# Development and evaluation of a tailored e-self-management intervention (dr. Bart app) for knee and/or hip osteoarthritis: study protocol

**DOI:** 10.1186/s12891-019-2768-9

**Published:** 2019-08-31

**Authors:** Tim Pelle, Karen Bevers, Job van der Palen, Frank H. J. van den Hoogen, Cornelia H. M. van den Ende

**Affiliations:** 1 Department of Rheumatology, Sint Maartenskliniek, Nijmegen, the Netherlands; 2 Department of Rheumatology, Radboud University Medical Center, Nijmegen, the Netherlands; 3 Department of Research Methodology, Measurement, and Data-Analysis, Behavioural, Management and Social Sciences, University of Twente, Enschede, The Netherlands; 4 Medical School Twente, Medisch Spectrum Twente, Enschede, the Netherlands

**Keywords:** Osteoarthritis, eHealth, Knee and/or hip, OA, Behavioural change techniques, Chronic disease, Machine learning

## Abstract

**Background:**

This paper describes (the development of) an eHealth tool (dr. Bart app) to enhance self-management and to optimize non-surgical health care utilization in patients with knee and/or hip osteoarthritis (OA) and presents a study aiming 1) to study the effectiveness of the dr. Bart app on health care use 2) to explore differences in use, usability and the clinical outcomes of the dr. Bart app between the Netherlands and Germany.

**Methods:**

The dr. Bart app is a fully automated eHealth application and is based on the Fogg model for behavioural change, augmented with reminders, rewards and self-monitoring to reinforce app engagement and health behaviour. The dr. Bart app propose goals to a healthier lifestyle based on machine learning techniques fed by data collected in a personal profile and choosing behaviour of the app user. Patients ≥50 years with self-reported knee and/or hip OA will be eligible to participate. Participants will be recruited in the community through advertisements in local newspapers and campaigns on social media. This protocol presents a study with three arms, aiming to include 161 patients in each arm. In the Netherlands, patients are randomly allocated to usual care or dr. Bart app and in Germany all patients receive the dr. Bart app. The primary outcome of the first research question is the number of self-reported consultations in secondary health care. The primary outcome of the second research question (comparison between the Netherlands and Germany) is self-management behaviour assessed by the patient activation measure (PAM-13) questionnaire. Secondary outcomes are costs, health-related quality of life, physical functioning and activity, pain, use and usability of the dr. Bart app. Data will be collected through three online questionnaires (at baseline and after 3 and 6 months after inclusion).

**Discussion:**

This study will gain insight into the effectiveness of the dr. Bart app in the (conservative) treatment of patients with knee and/or hip OA and differences in the use and usability of the dr. Bart app between the Netherlands and Germany.

**Trial registration:**

Dutch Trial Register (Trial Number NTR6693 / NL6505). Registration date: 4 September 2017.

## Background

Osteoarthritis (OA) is the most common joint disease in the world [1], affecting approximately 10–18% of the population aged 60 years and over. Most often hip and knee joints are affected by OA [2]. Osteoarthritis causes functional disability as a consequence of major structural changes of the joint (i.e. progressive loss of articular cartilage) and has a major impact on the quality of life [2–5]. As a result, the societal burden of OA is high; the annual burden of OA is 1.6% of the total health care expenditure in the Netherlands [6]. In the Netherlands, health care costs attributable to OA spent in secondary care are eight times higher than costs spent in primary care [6]. As the prevalence of OA increases with age [2, 7], it is expected that the burden of OA will increase dramatically in the near future.

Although OA is not curable, a variety of treatment options is available to reduce symptoms [8, 9]. Core elements comprise education, promotion of lifestyle changes (physical activity (PA)), pain management, exercise therapy, and weight reduction in case of overweight. Although total joint arthroplasty (TJA) is considered a (cost-)effective treatment for people with OA, TJA should only be considered after conservative treatments have failed [10–12]. Since OA is a chronic disease, a key element in the non-surgical management of knee and/or hip OA is self-management [5, 10, 11, 13]. Self-management interventions offer patients guidance in improving their skills to take care of themselves and to improve skills to navigate the health care system [14, 15].

Despite recommendations about the content of non-surgical treatment options in OA, quality of care in OA in primary care is suboptimal [12, 16, 17]. Lack of time and detailed guidance in clinical practice result in underutilization of non-surgical treatment options and (unnecessary) referrals to secondary health care in people with OA [10, 12]. Therefore, it is of importance to promote self-management in people with OA to optimize the use of non-surgical care and to prevent unnecessary referrals to secondary care.

Health education and goal setting should be considered as fundamental elements of (effective) self-management interventions [18–21]. Health education should include education about OA and its treatment options, pacing of PA and exercise, weight loss (if applicable) and how to find and utilize resources [10, 11, 15]. This information should be tailored to the person’s illness perception and educational capability [10]. Additionally, goal setting is a widely used behavioural change technique in many fields, especially in health care [21]. Goal setting is associated with positive effects on behaviour at both short and long term [21, 22]. Monitoring of behaviour or outcome (e.g. amount of PA, weight and achievement of goals), providing direct feedback and getting rewards may augment the effects of goal setting [21]. Traditional self-management programmes for OA show small benefits on self-management skills, pain, function and symptoms compared to usual care [23]. Ultimately, effective self-management implies that patients are able to take better care of their illness and, consequently, make optimal use of primary and secondary health care options.

Quality of care for people with OA is suboptimal and varies among (European) countries [17]. It is conceivable that differences in health care policies and cultural differences in health behaviour related to self-management account for differences in quality of care and clinical outcomes [24]. For example, in the Netherlands, the general practitioner (GP) functions as a gatekeeper while in Germany secondary care (e.g. rheumatologist and orthopaedic surgeon) is directly and more easily accessible to patients [25]. Dutch GPs tend to give more information and advice about a disease than their German colleagues in a consultation [26]. In addition, variations in culture could influence internet use and thus information seeking behaviour [24, 27]. These differences among countries may affect the way people with OA navigate the health care system [15, 28–30]. To our knowledge, there is limited insight in the differences in use and effects of (e-)self-management interventions among countries.

Modern persuasive technologies (e.g. applications) offer the possibility to enhance goal setting and provide tailored information to people with OA that suits individual preferences and to enhance self-management at all times [31, 32]. Moreover, modern technologies can monitor health behaviour and provide real-time feedback, which are considered important elements of self-management [15, 33]. Although the use of modern technologies seems promising, the majority of eHealth applications have not proven their effectiveness in clinical trials [34–38], especially in the field of OA. Therefore, we iteratively and systematically developed a standalone e-self-management application (dr. Bart app) in both Dutch and German language, incorporating education, setting achievable health behaviour goals and provision of feedback. This paper describes the design of a study that aims 1) To evaluate the short term effects (after 3 & 6 months) of use of the dr. Bart app in terms of (self-reported) number of consultations in secondary health care due to OA of the knee and/or hip in the Netherlands. We hypothesize that the app is (cost-)effective compared to usual care. 2) To explore differences in use, usability and clinical outcomes between the Netherlands and Germany. This paper also describes the systematical and iterative development of the dr. Bart app.

## Methods

### Development of the dr. Bart app

**Fig. 1:**
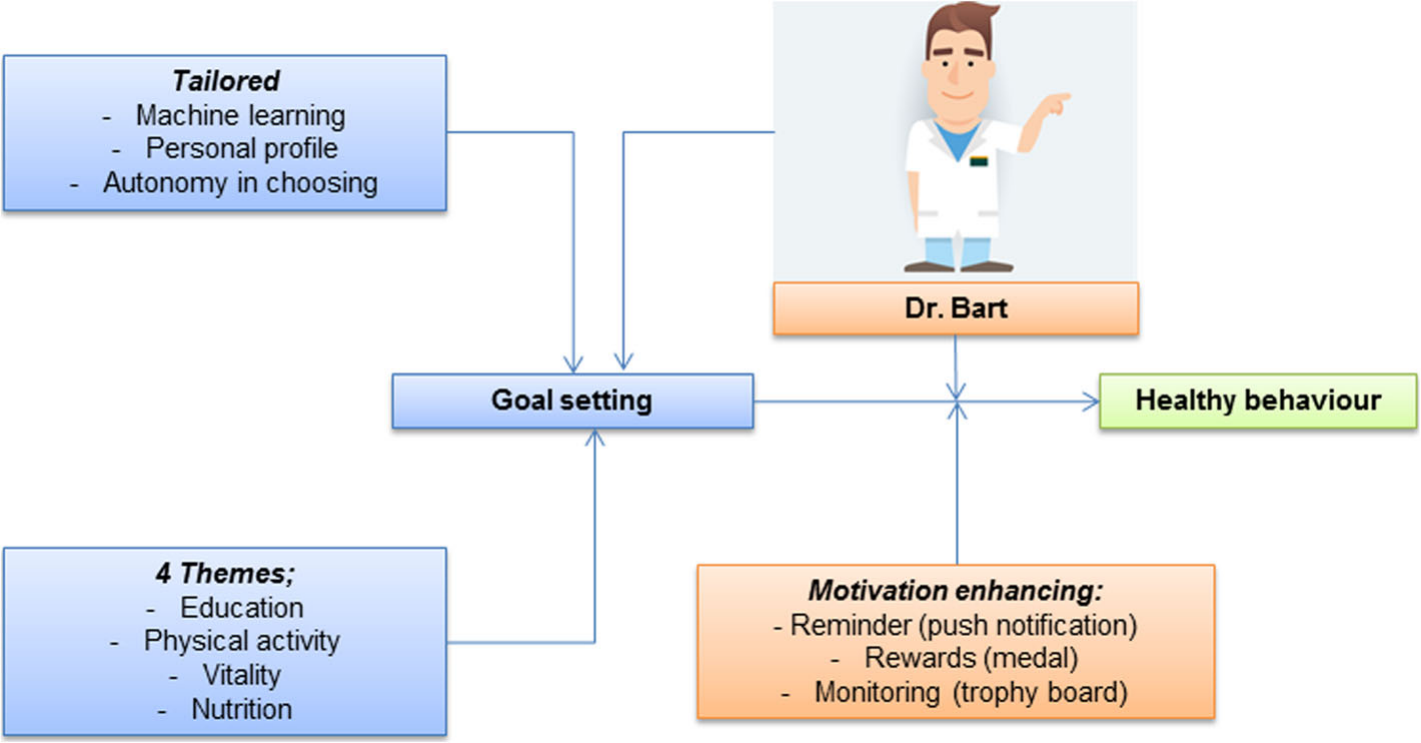
Theoretical framework of the dr. Bart app

We developed the dr. Bart app to enhance self-management and to actively involve people with OA in managing their disease. Prior to the development of the app, a project group of experts consisting of (medical) researchers, physicians, physical therapists, patient representatives and app developers (including a user experience expert) was installed. This project group decided upon the theoretical framework (Fig. 1), starting points and elements to be incorporated in the app. A model of behavioural change for the dr. Bart application was proposed based on the Fogg’s behaviour model (FBM) [39] and augmented with other motivation enhancing techniques (i.e. reminders, rewards and self-monitoring), that will help users to achieve goals and in the long run result in better lifestyle behaviour and ultimately health (Fig. 1) [22, 39, 40].

The steps followed during the different development phases of the dr. Bart app are elaborated upon in detail below. First, the applied theoretical framework of this standalone e-self-management intervention and its components are described and justified. Secondly, the iterative process of the development of the dr. Bart app is described.

### Theoretical framework of the dr. Bart app

Considering the fact that behavioural change is difficult to achieve, the project group decided to base the application on the widely used Fogg model for behavioural change, also known as the ‘tiny habits method’. [39] It states that three elements must be present for the target behaviour to happen: People must 1) Be (sufficiently) motivated. 2) Have the ability and 3) Be triggered, or reminded to perform the behaviour [39]. The FBM states that there is a trade-off between these 3 factors; motivation and ability must occur at the same moment at a given trigger, otherwise, the behaviour will not happen. A trigger is referred to as an event in daily life that elicits the behaviour. According to FBM, people with low motivation need an easy objective and a simple trigger to be motivated to perform a target behaviour. Rather than stating “I want to lose five kilograms within one month”, FBM suggest to establish tiny healthy habits. Thus, an objective could be “I eat one apple instead of unhealthy snack during my lunch break”. The accumulation of such small behavioural lifestyle changes results in the achievement of an overarching target behaviour (e.g. weight reduction) and ultimately better health.

#### Goal setting

The project group decided after review of the literature and consensus meetings that users should have the option to choose goals related to four main themes that are core elements in the (non-surgical) management of OA; 1. education regarding OA, its treatment modalities and the benefits of a healthy lifestyle 2. Physical activity (both generic and OA specific information), 3. Vitality, and 4. Nutrition. To facilitate goal setting by the user, a library of pre-formulated goals with suggestions for triggers was incorporated in the dr. Bart app. Moreover, the project group agreed that machine learning techniques should be used to propose goals tailored to the personal situation and needs of users and taking into account the user’s history of already achieved and discarded goals.

#### Tailoring goal setting

As motivation and ability are dynamic processes and vary among individuals in real life, dr. Bart uses machine learning techniques based on a recommender system to propose (tailored) pre-formulated goals to users that suits the motivation and ability of each user. A recommender system is a general term for algorithms concerned with providing recommendations to users [41]. One of the most well-known examples is found in web shops like Amazon. These web shops provide suggestions of products to buy based on earlier purchases and contextual information about their users. For the dr. Bart app the machine learning comprises a dynamic model (Contextual Multi-armed Bandit approach) proposing goals that will be challenging, achievable and tailored for that specific user. The machine learning will be fed with contextual and personal information collected in a personal profile [42].

To generate a personal profile, dr. Bart asks some relevant questions the first time users log in; name, gender, year of birth, length, weight, localization of symptoms, maximum walking distance and quality of sleep. Moreover, during the intake a user can select a preference of categories of goals he/she would like to work on. This preference is taken into account when making the initial recommendations for goals.

In order to give users autonomy in choosing goals, two techniques are incorporated in the dr. Bart app. First, users will be provided with the ability to tailor goals to their preferences by adapting both the level and trigger of a given pre-formulated goal. Second, the recommender system of the dr. Bart app proposes five pre-formulated goals to the user. Subsequently, the user can select or discard a goal to work on the next 24 h. Users can work on up to three goals simultaneously in the app.

#### Motivation enhancing techniques

The project group agreed to incorporate a combination of techniques in the dr. Bart app (i.e. reminders [43, 44], rewards [43, 44] and self-monitoring of behaviour [40]) to enhance motivation and to reinforce app engagement and, thus, augment the potential intervention effect. In addition, the project group embraced the idea to incorporate an authoritative and approachable character (dr. Bart cartoon) to address the needs of users for reliable information and advice [45–47] and to mimic personal interaction. Positive feedback by dr. Bart is provided by positive gestures and positive expressions (e.g. “well done!)”.

#### Reminders

After installing the app, users will receive a daily push notification from dr. Bart. This push notification is twofold; first, it reminds the user of the chosen goals for that day: “do you think of your goals today” and second, the push notification contains an interesting fact or a frequently asked question with an answer, or “Did you know that” about OA [48]. Additionally, the app automatically sends a push notification stating: “we have not seen you in a while, do you think of your goals?” when a user has not opened the app for more than 7 days.

#### Rewards

The dr. Bart app provides different types of rewards to users. By means of performance feedback, dr. Bart compliments the user: “Well done, 2 more goals to go for today”. Additionally, after a user checks the box that he/she achieved a goal, confetti appears on the screen and dr. Bart says for example “Well done, you achieved all of your goals today”. In a predetermined sequence, users can earn achievements. For instance, an individual can earn a bronze medal after achieving the same goal five times. Subsequently, a gold medal can be earned after 21 times of achieving a goal.

#### Monitoring

An overview of achieved goals and how often these goals are achieved is presented in the “my goal page”. A trophy cupboard in the app is used to illustrate the individual progress, i.e. earned achievements.

### The development process of the app

Prior to the actual development of the dr. Bart app, a list of starting points was formulated by the project group to establish stakeholders’ most important needs and functionalities in the use of the app based on the described theoretical framework.

#### Iterative design process

Both the (graphical) design and the content of the dr. Bart app were developed in an iterative design process with sprints of 3 weeks. Each sprint consisted of development, (user-)testing, adaptation, re-testing and final design [44, 49]. Applications that are developed according to the methodology of persuasive design result in better treatment adherence compared to other techniques [50].

#### Development of the (graphical) design of the dr. Bart app

First, user stories regarding user requirements were developed by the app developer based on the list of starting points and input of the project group for the different components of the app (e.g. personal profile, library of goals, education library). First step in the development was the delivery of mock-ups (graphical design) for the different components of the app. In each mock-up round for the graphical design, 5–10 participants (including members of the project group) gave suggestions for improvements and alterations were made. This process was repeated until no further adaptations were deemed necessary for the graphical design. Subsequently, the actual screens were developed and a blueprint for navigation was created (wire-frame). This wire-frame was iteratively tested until no further adaptations were deemed necessary resulting in a beta version of the dr. Bart app.

#### Development of content

Parallel with the (graphical) design process the content of the dr. Bart app was iteratively developed.

#### Formulation of goals

The pre-formulated goals used in the dr. Bart app were formulated in co-creation with physical therapists, physicians and patient representatives. First, 4 members of the project group each delivered a list of 30 goals relevant for the treatment of OA and fulfilling the SMART criteria (Specific, Measurable, Achievable, Relevant and Time-bound) [18]. These 4 lists were merged and duplicates were removed by the first author (TP), resulting in a list of 72 goals. Subsequently, the goals of this new list were rephrased by the first author (TP) so that the goals require low motivation and ability (tiny habits) [39].

#### Education of OA

In the educational library of the dr. Bart app specific information regarding OA, and generic lifestyle advice can be found. Moreover, Frequently Asked Questions (FAQs) are part of the educational library of the dr. Bart app. The answers to these FAQs are thoroughly considered by an expert group [48]. In addition, information about the themes (i.e. physical activity, vitality and nutrition) is given in the educational library.

#### Physical activity and exercise library

To facilitate exercise and PA the dr. Bart app contains generic information on PA and its positive influences on health. In addition, an exercise library is incorporated in the dr. Bart app. Four physical therapist specialized in the treatment of OA each delivered a list of ten exercises which they considered important and were easy to execute without supervision. These lists were combined by the first author (TP) and duplicates were removed resulting in a list of 21 different exercises. After a consensus meeting with all involved physical therapists and the project group, a list of 14 exercises remained, focusing on both strengthening and flexibility of the lower extremity. These 14 exercises are illustrated in an exercise library by means of animated Graphics Interchange Format (GIF). These GIFs are accompanied with textual information on how to perform the specific exercise.

#### Vitality

For people with OA it is important to learn to pace their activities. Therefore, the dr. Bart app contains generic information regarding pacing of activities and vitality (e.g. sleep quality) and its positive influences on health and OA symptoms.

#### Nutrition

To enhance a healthy lifestyle, the dr. Bart app contains generic information regarding nutrition and its positive influences on health and OA symptoms. Goals regarding nutrition will target on weight management and healthy behaviour (e.g. “today I eat an apple rather than an unhealthy snack” or “today I do not drink alcoholic beverages”).

#### Pilot test of the dr. Bart app combined with training of the machine learning

The beta version of the dr. Bart app was pilot tested on usability in a sample of the target population. Machine learning has a “cold start”: at the beginning the recommender system does not know anything about the relation between the goals because it has no history of users picking goals. To reduce this issue and to make sure that the first users get recommendations that are sensible, the system is bootstrapped by providing it domain knowledge about the goals.

In order to “train” the machine learning, 25 persons with knee and/or hip OA with the same characteristics as the target population consented to pilot test the dr. Bart app for 1 month. Simultaneously, the feasibility and usability of the dr. Bart app were evaluated in the same pilot group. Moreover, we invited 5 participants from the pilot group for a user experience session with a semi-structured group interview, led by a user experience expert. In addition, we invited 5 people with equal sociographic characteristics as the target population to use the app for the very first time, including downloading the app, while making use of the “thinking-aloud” principle [49]. Based on the usability test, final alterations were made (version number 1.3.7, end of 2017).

#### Translation process of the dr. Bart app

The second aim of the described study is to explore potential (cultural) differences in use of the dr. Bart app and its effect on clinical outcomes between the Netherlands (arm B) and Germany (arm C). Therefore, the final version of the app and its content was translated to the German language by an independent native non-medical German speaker. All German texts were reviewed by an independent German communication adviser, one non-medical involved person, a medical doctor and a physical therapist. Consequently, small alterations to the German texts were made.

### Study design

**Fig. 2:**
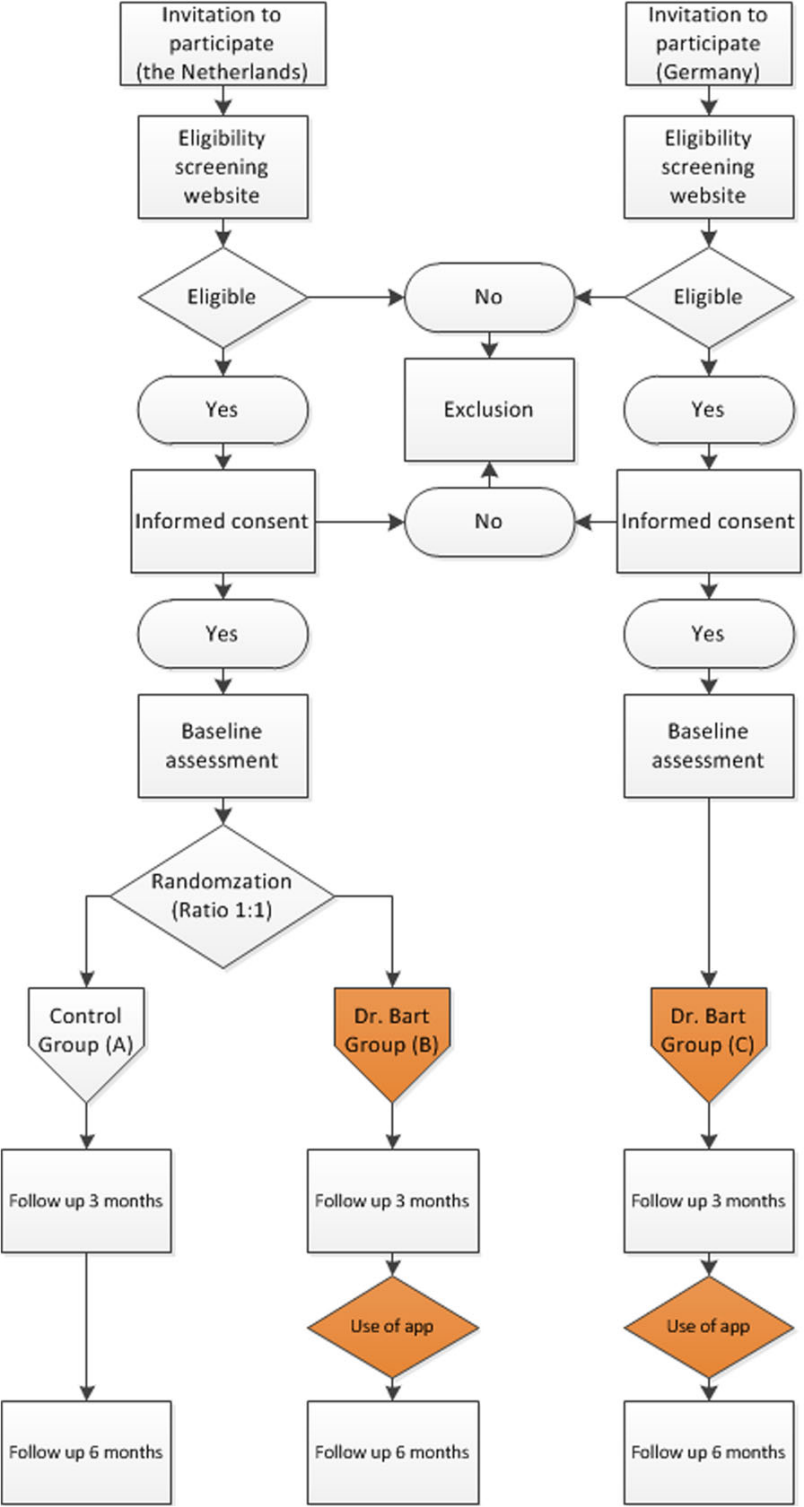
Flowchart of the study design with two arms in the Netherlands (A + B) and one arm (C) in Germany

This manuscript was reported according to the SPIRIT statement [51]. This is a three-armed study comprising a two-armed unblinded randomized controlled trial (RCT) in the Netherlands and a controlled clinical trial (CCT) in Germany and the Netherlands in patients with OA (Fig. 2). The RCT comprises a usual care group (group A) and a group receiving the dr. Bart app (group B). In addition, a third arm consisting of only German participants will all receive the dr. Bart app (group C).

### Participants

#### Inclusion criteria

In order to be eligible to participate in this study, a subject must meet all of the following criteria:

- Self-reported OA of the knee and/or hip, defined as:
  - Having a painful knee and/or hip
  - **AND -** Knee and/or hip pain > 15 days of the past month
  - **AND -** Morning stiffness < 30 min (knee) and/or < 60 min (hip)
- ≥ 50 years
- Having an email address
- Possession of smartphone or tablet and willing to download the dr. Bart app on one or more devices.
- Able to read, write and sufficiently communicate in Dutch or German language, where appropriate.

#### Exclusion criteria

Potential participants will be excluded from participation in this study if they meet any of the following criteria:

- Being wheelchair-bound
- Total joint arthroplasty of the knee and/or hip in the past
- Scheduled for knee and/or hip total joint arthroplasty in the next 6 months
- Diagnosis of (other) inflammatory rheumatic disease

### Study procedure

#### Recruitment and screening procedure

This study will be conducted by two hospitals, i.e. Sint Maartenskliniek Nijmegen (the Netherlands) and Sankt Elisabeth-Hospital Meerbusch (Germany). All participants for the described study will be recruited in the community through advertisements in local newspapers (i.e. region Nijmegen in the Netherlands and Meerbusch in Germany) and in campaigns on social media of the involved hospitals (i.e. Facebook, LinkedIn, Twitter). Individuals with OA in the community willing to participate will be invited to visit the website www.drbart.eu and to complete a number of questions to check their eligibility for participation in this study. Eligible individuals are asked to sign in for the study by providing their e-mail address on the website. Hardcopy Information regarding the described study and generally on scientific research will be sent to their home address on demand. Potential participants will receive online baseline assessment via CastorEDC (https://www.castoredc.com/). CastorEDC is an electronic software application for data collection and management. CastorEDC is approved with ISO 27001 and ISO9001, and is in line with the EU Data Protection Directive.

#### Randomization, allocation concealment and blinding

Dutch consenting participants will be randomly allocated by the researcher to usual care (A) or dr. Bart (B) (allocation ratio 1:1) after completing baseline assessment performed with CastorEDC. CastorEDC uses a validated variable block randomization method (with randomly varying block sizes of 2, 4 and 6) and is stratified on the individual level by hip or knee OA. The researcher who will ascertain randomization will be concealed for treatment allocation. Due to the design of this study, blinding of participants and researchers is not possible. Data collectors will be blinded as data will be collected with validated questionnaires via CastorEDC. All German participants receive the dr. Bart app during the study (C), see Fig. 2.

#### Intervention (dr. Bart app)

Participants allocated to the Dutch dr. Bart group (B) and all German participants (C) will receive the dr. Bart app during the study.

#### Experimental intervention groups (arms B and C)

Participants allocated to the Dutch intervention group (B) and all German participants (C) will receive the dr. Bart app (version number 1.3.7) for 6 months. They can use the dr. Bart app “ad libitum”. The app is only accessible for users after the researcher has provided access for the app. Throughout the study, participants are able to call and send emails to the researcher when they have questions regarding the dr. Bart app or the study.

#### Usual care control group (arm A)

Half of the participants in the Netherlands will be allocated to the usual care group (arm A) and will receive no active treatment, but care as usual. Usual care is defined as de facto clinical care and will not interfere with usual care.

### Measurements

Three self-assessed sets of online questionnaires (at baseline and, 3 and 6 months after inclusion) will be collected with CastorEDC, (Table 1) and, where applicable, a reminder will be sent after 1 week. Participants will not receive (financial) incentives or other compensation for completion of the questionnaires or the study.

**Table 1:** Assessments in the Netherlands (arm A + B) and in Germany (arm C) at baseline and at follow-up of 3 and 6 months

	Baseline assessment (T0)	Follow-up 3 months (T3)	Follow-up 6 months (T6)
Patient characteristics (A + B + C)	X		
Cost questionnaire (A + B)	X	X	X
PAM-13 (A + B + C)	X	X	X
EQ-5D-3 L (A + B + C)	X	X	X
HOOS/KOOS (A + B + C)	X	X	X
SQUASH (A + B + C)	X	X	X
TOA (A + B + C)	X	X	X
IPQ-K (A + B + C)	X	X	X
OA-QI (A + B + C)	X	X	X
SUS (B + C)		X	X
Use of app (B + C)		X	X

*Abbreviations*: *PAM-13*, Patient activation measure, *EQ-5D-3 L* Euro Quality of Life – 5 Dimensions - 3 Level, *HOOS/KOOS* Hip/Knee Osteoarthritis Outcome Score, *SQUASH* Short Questionnaire to assess health-enhancing physical activity, *TOA* Treatment beliefs in knee and hip osteoarthritis, *IPQ-K* Illness Perception Questionnaire, *OA-QI* Osteoarthritis Quality Indicators, *SUS* System Usability Scale

### Study parameters

#### Main study parameter

The main study parameter for the RCT in the Netherlands (arm A vs. B) is the number of self-reported consultations in secondary healthcare (i.e. orthopaedic surgeon, rheumatologist, physician assistant) due to OA in the knee and/or hip in the past 6 months, collected every 3 months. The primary endpoint in the Netherlands is the difference in mean consultations in secondary health care at 6 months between the usual care group (A) and the dr. Bart app group (B).

The primary endpoint for the comparison between the Dutch dr. Bart app group and the German dr. Bart app group (arm B vs. C) is self-management behaviour, as collected with the Patient Activation Measure (PAM-13) questionnaire [52, 53] at 6 months.

#### Secondary study parameters

Secondary study parameters include direct medical costs, self-management behaviour, health-related quality of life, physical function (in daily living, sport and recreation), physical activity, treatment beliefs in OA, illness perception, perceived quality of care, and use and usability of the dr. Bart app.

### Assessments

#### Direct medical costs

By means of an OA specific questionnaire direct medical costs (i.e. healthcare-related) due to OA in the 6 months of follow-up will be collected, in both secondary and primary healthcare. Standard cost prices of the Dutch costing guideline will be used [54]. Direct medical costs will be computed by multiplying the cost prices with the usage frequency as reported by participants.

#### Self-management behaviour

The PAM-13 questionnaire measures knowledge, skills and confidence to cope with one owns health [55]. The PAM-13 questionnaire is scored on a 13 to 52 scale. A higher score indicates higher level of patient activation. The PAM-13 shows good psychometric capabilities for measuring patient activation [53].

#### Health-related quality of life

EQ-5D-3 L will be used to measure health-related quality of life. This instrument contains questions about; mobility, self-care, usual activities, pain/discomfort and anxiety/depression, scored on a 3-point Likert scale. Moreover, the EQ-5D-VAS (visual analogue scale) will be used to indicate health-related quality of life on a vertical line, ranging from 0 (worst imaginable health) to 100 (best imaginable health) [56, 57].

#### Physical function in daily living, sport and recreation

Pain, symptoms, function in daily living, function in sport and recreation and knee or hip related quality of life in the previous week will be assessed with the Knee or Hip Osteoarthritis Outcome Score (KOOS or HOOS), depending on the affected joint [58, 59]. We will report subscales, since the total score is not validated. A higher score on the subscale indicates less complaints on that domain.

#### Physical activity

Physical activity will be assessed with the Short Questionnaire to Asses Health-enhancing physical activity (SQUASH). The SQUASH is a structured questionnaire consisting of activities at work, commuting, household activities, leisure-time and sports activities [60]. From this questionnaire time spent in light, moderate and vigorous physical activity can be calculated. The SQUASH is considered a reasonably valid tool to assess PA [60, 61].

#### Treatment beliefs in OA

The Treatment beliefs in osteoarthritis (TOA) questionnaire will be used to assess participants beliefs about various treatment modalities in hip and knee OA and is based on the theory of planned behaviour. Both positive and negative beliefs regarding five treatment modalities in OA are assessed; 1) Physical activities, 2) Pain medication, 3) Physical therapy, 4) Injections, 5) Joint replacement surgery. For each subscale items are measured on a 5-point Likert scale (1 ‘disagree to 5 ‘agree’). Psychometric properties are satisfactory to good [62]. The TOA is not yet available in German language. Therefore, we will translate and culturally adapt the TOA to the German language according to the forward and back translation principle [63].

#### Illness perception

Illness perception will be assessed with the brief illness perception questionnaire [64]. This is a short questionnaire consisting of eight dimensions of illness perceptions which can be scored on a 10 point scale. From these eight dimensions a total score can be calculated, where a higher score reflects more threatening views regarding OA than a lower score. An additional open question asks the three perceived most causative factors for an individual’s hip and/or knee OA.

#### Quality of care

A questionnaire regarding quality indicators for OA care is used to assess quality of care [65]. This questionnaire is available in Dutch, but not yet validated. For the German variant, we will translate and culturally adapt the English OA quality indicators questionnaire to the German language according to the forward and back translation principle [63].

#### Usability of the app

The usability of the app will be assessed with the System Usability Scale (SUS) [66, 67]. This questionnaire contains 10 questions regarding usability of the app. A total score ranging from 0 to 100 is calculated, where a higher score indicates better usability. Additionally, we provided a free-text opportunity after each question so that participants could elaborate on their given answers.

#### Adherence to the dr. Bart app

Adherence to the dr. Bart app will be quantitatively measured in the back end of the app. Parameters of use will be logged and extracted automatically for each user:

- *The proportion of active users (*e.g. *who completed minimal one goal) over time*
- *Number of average logins per active user per week*
- *Number of recommended goals, not chosen*
- *Number of chosen goals*
- *Number of achieved goals*
- *Number of goals set to non-active (*i.e. *choose to not do goal anymore)*
- *Number of goals set to active (*i.e. *choose to do goal again)*
- *Ranking of goals on the basis of times chosen*

#### Other assessments

Socio-demographic characteristics (i.e. age, gender, length, weight, localization and duration of OA symptoms, marital status, living situation, education, ethnicity, and (paid) work) will be collected at baseline.

### Sample size

The primary outcome measure for the RCT is number of consultations in secondary health care due to OA of the knee and/or hip in the past 6 months. We performed a sample size calculation for an unpaired t-test to compare two independent means. Based on previous research a mean difference of 0.35 consultations between two independent groups was considered credible, with a standard deviation (SD) of 1.00 [68]. With a power of 0.80 and an alpha (α) of 0.05 (two-sided) a sample size of 129 participants per arm is needed. Accounting for loss to follow-up of 20%, 161 participants per arm will be included. Sample size was calculated with Stata version 13.1.

The second aim of the presented study is to explore cultural differences in use and usability of the app and its effect on clinical outcomes. For pragmatic reasons, we decided to include 161 participants (including 20% loss to follow-up) as well in the German arm of the study (C). A total of 129 participants is sufficient to detect a minimal difference of 4.6 (SD = 13.2) on a 52 point scale in the PAM-13 questionnaire between the Dutch (B) and German (C) groups (all using the dr. Bart app) with a power of 0.80 and an alpha of 0.05 (two-sided). The target sample will be able to detect a small to medium effect size (0.2–0.4) in primary outcomes (i.e. number of consultations in secondary health care and self-management behaviour) between groups.

### (Planned) statistical analyses

All statistical analyses will be performed using Stata 13.1 (www.stata.com). For each questionnaire separately, (missing) data will be managed according to recommendations of the specific questionnaire. Descriptive statistics will be used to present group characteristics. Normality will be assessed for continuous data by checking histograms. Continuous variables will be reported as mean and SD or median and inter-quartile ranges, as appropriate. For nominal variables, number (N) and percentage (%) will be presented. The primary analysis will be performed according to the intention-to-treat principle. In addition, a per-protocol analysis, including adherent participants of the dr. Bart group and the entire usual care group will serve as sensitivity analyses. In order to check for selective attrition, baseline characteristics of completers and non-completers of the study will be compared. In all analyses, a two-tailed significance level of *p* < 0.05 is regarded as statistically significant. Differences between treatment arms at baseline will not be statistically tested.

### Efficacy

Differences in the primary and secondary outcomes between the two arms of the RCT in the Netherlands (A vs. B) after 3 and 6 months will be assessed by either linear mixed models or Poisson regression, as appropriate, with a random intercept, including treatment group (i.e. usual care (A) or dr. Bart (B)), baseline value and interaction between treatment group and time as covariates. The primary outcome in the Netherlands will be reported as an incidence rate ratio in case of Poisson regression, or negative binomial regression, as appropriate.

### The Netherlands vs. Germany

Differences in means, at 3 and 6 months of follow-up, between the Dutch dr. Bart group (B) versus the German dr. Bart group (C) will be explored by mixed model repeated measures analyses with a random intercept, including country (i.e. the Netherlands or Germany), accompanying baseline value and interaction between country and time. Analyses will be corrected for relevant confounders; age, gender and BMI among others.

### Use of the app and its relation with clinical outcomes

The relation between clinical outcomes and use of the app will be studied using multivariable regression analyses with clinical outcome (e.g. physical functioning, PA) as the dependent variable, whereas quantitative data about use of the app will serve as independent variable(s). We hypothesize that participants who more frequently use the dr. Bart app will improve more on clinical outcomes (e.g. physical functioning) compared to less frequent and non-users. Additionally, a Kaplan-Meier curve will be constructed to illustrate the proportion of persons who start using the app (i.e. who have chosen at least one goal) over time.

### Economic evaluation from the health care perspective

The economic evaluation is based on the general principles of cost-utility analysis (CUA) and cost-effectiveness analysis (CEA), applying a health care perspective. Direct medical cost in the Netherlands due to OA in the 6 months of follow-up will be analysed, in both primary and secondary care and will be measured retrospectively by online questionnaires on a 3 month recall basis. Standard cost prices of the Dutch costing guideline will be used [54]. To determine the incremental cost-effectiveness ratio (ICER) for the CUA, differences in costs between the usual care group (A) and the dr. Bart group (B) will be divided by differences in QALYs. For the CEA, differences in costs between the groups (A vs. B) will be divided by the differences in secondary outcomes (e.g. physical functioning, physical activity). Uncertainty (95% CI (confidence interval)) around the ICERs will be stochastically determined by the bootstrap method.

### Timeline

The development of the dr. Bart app was finished at the end of 2017. Recruitment of participants in the Netherlands is ongoing as of January 2018. Participants will be included until the required sample size is acquired. For the German part of the study, we will start recruiting participants as of June 2019.

## Discussion

As the prevalence of OA is expanding and costs related to OA care will increase, effective treatment modalities need to be developed for people with OA. The presented study will investigate the effect of an e-self-management intervention, equipped with machine learning techniques, on changing behaviour to improve health behaviour in people with knee and/or hip OA. This study comprises three arms; a usual care arm (A) and a dr. Bart app arm (B) in the Netherlands and one arm, all receiving the app, in Germany (C).

Our choice for the primary outcome in the Dutch part of the study (i.e. number of self-reported consultations in secondary health care) needs further explanation. Self-management interventions aim to increase the capacity of patients to cope with symptoms rather than controlling symptoms. Optimal self-management requires that the person understands the illness and manages their care, including skills navigating the health care system and apply these to take better care of themselves [15, 69]. In the literature there is no mutual agreement on the primary outcome assessing (e-)self-management interventions [23, 70]. Commonly used primary outcome measures for assessing effectiveness of self-management interventions are measures for pain, self-efficacy and physical functioning, but these measures do not reflect the ultimate aim of self-management; take care of one’s own health and improve skills to navigate the health care system and thus change health behaviour [70, 71]. In our view, change in health care utilization patterns reflects indeed a change in behaviour and is a valid proxy for self-management [48, 72].

As to the design of the study there are potential issues that need to be addressed. First, we include participants on the basis of self-reported knee and/or hip OA and this could result in a selective study population, and as a consequence reduce generalizability to the wider population. However, a meta-analysis showed that the self-reporting of OA results in acceptable diagnostic properties; sensitivity of 0.75 (95% CI: 0.56–0.88) specificity of 0.89 (95% CI: 0.77–0.95) [73]. Therefore, we assume that the inclusion on the basis of self-reported OA is appropriate.

Second, (non)adherence to eHealth applications is considered a problem and as a consequence the effectiveness of these interventions possibly diminishes. In the dr. Bart app, a variety of elements (e.g. reminders) is incorporated to reinforce app engagement and in turn augment intervention effects [43, 50]. Moreover, the dr. Bart app is a standalone software application without human interaction, while it has been shown that blended options have more impact on health outcomes [50]. We assume, however, that the machine learning will provide tailored guidance and therefor better adherence to the treatment of OA.

Several strengths need to be underlined. The theoretical framework of the dr. Bart app is based on a solid rationale and the incorporated behavioural change techniques are chosen by specialists from different fields. In addition, all elements of the dr. Bart app are developed in co-creation with patient representatives and specialists. The design process of the dr. Bart app was based on an iterative design process resulting in a beta version which was pilot tested during a month in 21 people with OA. This is the first study that examines potential differences in use, usability and clinical outcomes of an e-self-management application for people with OA between the Netherlands and Germany.

In conclusion, this study will gain insight in the effectiveness of a standalone software application (dr. Bart app) equipped with machine learning techniques in the (conservative) treatment of people with knee and/or hip OA. Additionally, this study provides information regarding (cultural) differences in the (conservative) treatment of OA between the Netherlands and Germany.

### Patient involvement

Patient representatives from the Netherlands actively collaborated with researchers in a project group during the entire iterative design process of the dr. Bart app, as presented in our methods section. They were involved in the choice for the theoretical framework, formulation of goals, choice of the applied behaviour change techniques, iterative development of the (graphical) design, content, user experience session and pilot test among other*s.*

## Data Availability

Data sharing is not applicable to this article as no datasets were generated or analysed for this manuscript.

## Abbreviations

BMI: Body Mass Inde*x*
*COPD*: *Chronis Obstructive Pulmonary Disease*
*DM*: *Diabetes Mellitus*
OA: Osteoarthritis
PA: Physical activity
*RA*: *Rheumatoid Arthritis*
SMK: Sint Maartenskliniek Hospital
TJA: Total joint arthroplasty
*WHO*: *World Health Organization*
